# Surgical treatment of colon cancer: Colentina surgical clinic experience

**Published:** 2012-09-25

**Authors:** B. Mastalier, C. Tihon, B. Ghita, C. Botezatu, V. Deaconescu, P. Mandisodza, C. Draghici, S. Simion

**Affiliations:** General Surgery Clinic, Colentina Clinical Hospital Romania

**Keywords:** colonic cancer, occlusion, anemia, diastatic perforation, hemicolectomy, segmental colectomy

## Abstract

Most patients with colon cancer are surgically treated, with postoperative association of chemotherapy and possibly immunotherapy in advanced cases. Surgical treatment is chosen depending on the evolution stage, tumor topography and the existence of complications, colonic surgery being dictated by colonic vascularization. The radical character of the surgical intervention can be assured only in the early stages of the tumor. Colostomy is rarely necessary in patients with colon cancer. In the period of the last 5 years (2007-2011), 307 patients with colon cancer were operated in “Colentina” Surgical Clinic, radical intervention being possible only in 219 cases. 48 cases were emergency interventions for occlusion or perforation with peritonitis. Every time the mechanical preparation of the bowel was not possible, the intraoperative washout technique was used. Postoperative complications were met in 27 cases, being represented by bleeding (3 cases), peritoneal abscess (5 cases), anastomotic fistula (7 cases), abdominal wound infection (12 cases). In 5 cases the operation was done laparoscopically. Preoperative mortality was of 13 cases. Postoperative chemotherapy was done in the great majority of cases.

## Introduction

Situated in the fourth place in the world regarding neoplazic frequency, colon cancer is responsible for 10% of the malignant tumors in the European Union, having the 3rd position among the digestive cancers, with a relative equality regarding the sex ratio, the highest incidence being met in the 6-7 decades of life [**1-6**]. The topography is represented in 60% of cases by left colonic localization (among them 75% is only sigmoid topography), the rest of 40% being represented by right colon tumors; in 5% of the cases multiple (synchronous) tumors are met. The etiopathogeny of colon cancer recognizes both an hereditary predisposal (hereditary non-polyposic colonic cancer, familial adenomatous polyposis, inflammatory nonspecific colonic diseases, cancer antecedents) and a favorable influence of medium factors (diet poor in cellulosic fibers and rich in animal fat, exposure to carcinogens of biliary acids type, alimentary additives, alcohol or ionizing radiation) [**7,8**]. The lately increase of colon cancer incidence in our country is due to the alteration of alimentary habits and life style of the people, more and more occidental, but also to the improvement of diagnostic means, with an increase of the diagnostic accessibility and better sanitary education of the population.

### General view

Macroscopic aspects of colonic cancer are represented by one of the following 3 types: vegetant tumors (almost always used for right colonic topography), ulcerative tumors (more frequently on the left colon, but sometimes associated with the vegetant form, due to the low intra-tumoral vascularization, leading to an ulcero-vegetant type) and infiltrative or stenosing tumor (almost specific for the left colon, with infiltration of the whole intestinal wall, on a limited distance - aspect of ligated bowel, respectively on a longer distance - aspect of rigid tube). Microscopic aspect of colonic cancer is represented in the highly majority of cases by adenocarcinoma with different degrees of differentiation (high, low, anaplastic), but colloid or mucous carcinoma (10-20%, often met in young, with high level of malignancy) or very rare mesenchymal tumors can also be met. Dissemination ways in colonic cancer are represented by continuity, contiguity, endolumenal, lymphatic, vascular and peritoneal ways.

In case of colon cancer, there are 2 staging systems with prognostic role in use:

- Astler-Coler classification, with subsequent stages: A (lesions limited to mucosa and sub-mucosa), B₁ (tumor that invades muscularis propria), B₂ (tumor invading all intestinal wall layers), C₁ (tumor confined to intestinal wall, with lymphonodes involving), C₂ (tumor that pass over the serosa, with lymphonodes involving), D (distance metastases);

- TNM classification, with T stages (Tx - primary tumor cannot be evaluated, T₀ - absent tumor, Tis - in situ carcinoma, T₁ - extension in submucosa, T₂ - invasion in muscularis propria, T₃ - extension to serosa and pericolic fat, T₄ - extension in adjacent organs, without fistula formation T4a or with fistula formation T4b), N stages (N₀ - without lymphnodes involvement, N₁ - invasion in 1-3 region lymphonodes, N₂ - invasion in 4 or more region lymphonodes, N₃ - involvement of distance lymphonodes, along the main vascular trunk) and M stages (Mx - presence of metastases cannot be evaluated, M₀ - without metastases, M₁ - with metastases).

**Table 1:** The equivalence between the different staging classifications:

stage	TNM	Astler-Coller	5 years survival rate
0 (in situ carcinoma)	Tis, N₀, M₀		100%
I	T₁, N₀, M₀	A	85%
	T₂, N₀, M₀	B₁	75%
II	T₃, N₀, M₀	B₂	65%
	T₄, N₀, M₀	B₃	55%
	T1-2,N1-3,M₀	C₁	45%
III	T₃, N1-3, M₀	C₂	35%
	T₄, N1-3, M₀	C₃	25%
IV	any T, any N, M₁	D	0%

The clinical picture of colonic cancer associates manifestations that can be seen below [**9,10**]:

- general manifestations: inappetancy, physical asthenia, weight loss, functional disorders such as disorders of intestinal transit (constipation or constipation-diarrhea alternation), abdominal pain, bleeding (melenic aspect in case of right colon cancer, red blood aspect in case of left colon cancer);

- manifestations specific for right colon cancer: anemic syndrome, right iliac fossa or right abdominal flank pain, intestinal transit disorders with abdominal distension in case of ileo-cecal valve tumor topography, palpable abdominal mass;

- manifestations specific for left colon cancer: intestinal transit disorders (progressive constipation or alternation with watery diarrhea, colitic dyspepsia with flatulence), pain localized in right iliac fossa (Bouveret syndrome preceding a diastatic perforation), stool aspect alteration (faeces with mucus and blood);

- borrowed manifestations of gastroduodenal or biliary type in case of transverse colon cancer.

Biologic exam can show: anemia, leukocytosis, thrombocytosis, inflammatory syndrome (increased levels for fibrinogen and VSH), hypoproteinemia, positive tumor markers (CEA, CA [**9**]). Imagistic investigations are represented by: colonoscopy with biopsy and histopathological exam (the only one that can sign the diagnosis for malignancy), irigography, plain abdominal radiography (in case of intestinal occlusion), abdominal echography possibly followed by CT-scan or MRI (better evaluation of the neighborhood relations and of locoregional spreading with the assessment of secondary lymphnodes or hepatic determinations or even with other localization, of possible ureteral obstruction with uretero-hydronephrosis), urography, pulmonary radiography (to discover possible lung metastases), exploratory laparoscopy (for staging of disease). In case of colon cancer, the metastatic involvement is represented in decreasing order by: liver (35%), lung (19%), retroperitoneum (13%), bone (4%), ovary (1-2%), suprarenal (1-2%).

**Figure 1:**
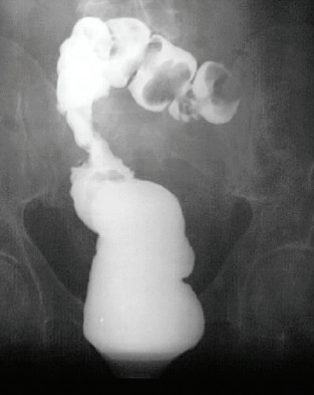
Tumor of hepatic flexure of colon - irigography

**Figure 2:**
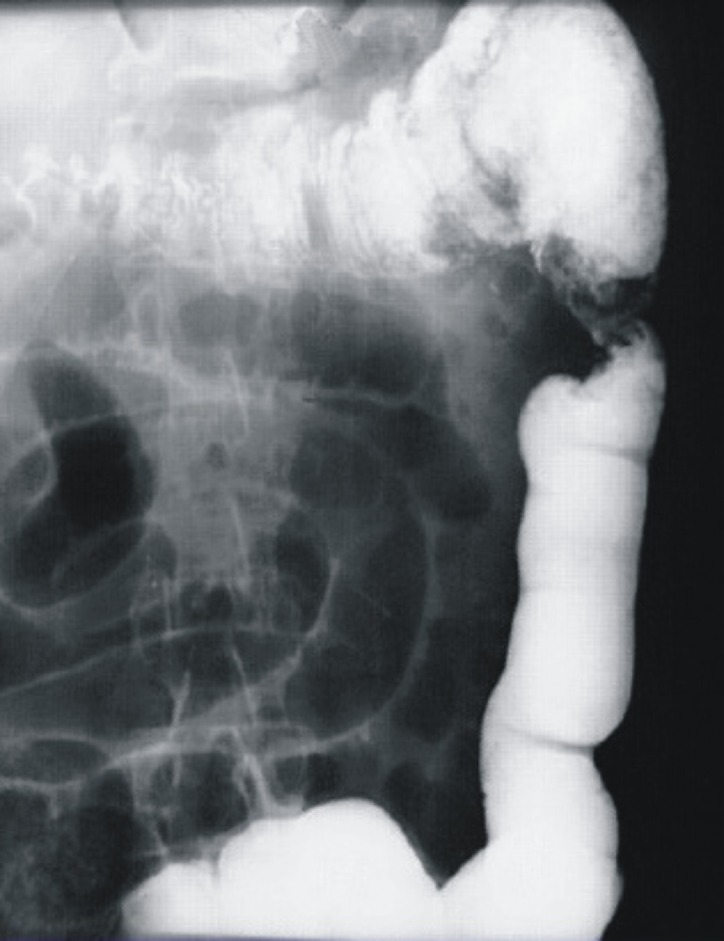
Tumor of splenic flexure of colon - irigography

**Figure 3:**
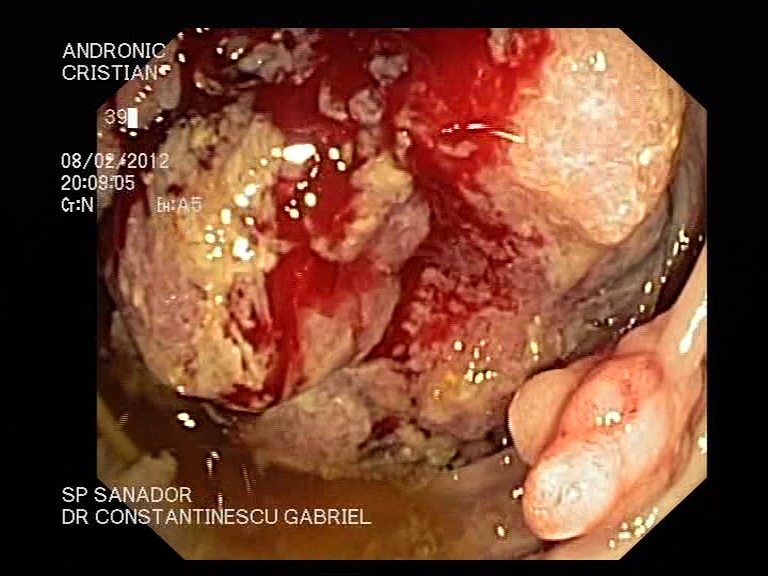
Tumor on cecum - ascending colon developed on familial adenomatous polyposis - endoscopic aspect

The diagnosis of colonic cancer in early stages, potentially curative by means of radical surgical treatment followed by adjuvant oncologic therapy is possible only by submitting population from risk groups to modern screening methods represented by tests of discovering the occult stool bleeding (Hemocult) and colonoscopy. Each subject around the age of 40 years, presenting intestinal transit alteration, dyspepsia or anemia, should be proposed for colonoscopy.

Differential diagnostic of colonic cancer could be made with other intestinal diseases generating of occult bleeding (hiatal hernia, benign or malign tumors, gastro-duodenal ulcer, diverticulitis, polyposis) or diseases presenting like abdominal palpable tumor (appendical plastron, cecal tuberculosis, Crohn disease, ulcerative colitis, retroperitoneal tumors etc.).

In case of absence of correct treatment, evolution of colonic cancer goes toward developing of dangerous complications (intestinal occlusion, localized or generalized peritonitis by tumor perforation or diastatic cecum perforation, fistulization to a neighbor organ - stomach, duodenum, small bowel) and finally to exitus.

Surgical treatment, determinant for the subsequent evolution, consists in radical interventions (right or left hemicolectomy, segmental colectomy on transverse or sigmoid colon, subtotal or total colectomy, with digestive continuity reestablished by ileocolic, colocolic, colorectal or even ileorectal anastomosis) or palliative (colostomy or colonic bypass in advanced non-resectable stages). Tumors with invasion in neighbor organs may lead to one block resections that associate distal splenopancreatectomy, gastrectomy, etc. Emergency operated cases usually demand a sequential solution, the initial time that remove the tumor and ends by colostomy being followed after a free interval of 3-6 months by a secondary time that reestablishes the digestive continuity [**11**].

**Figure 4:**
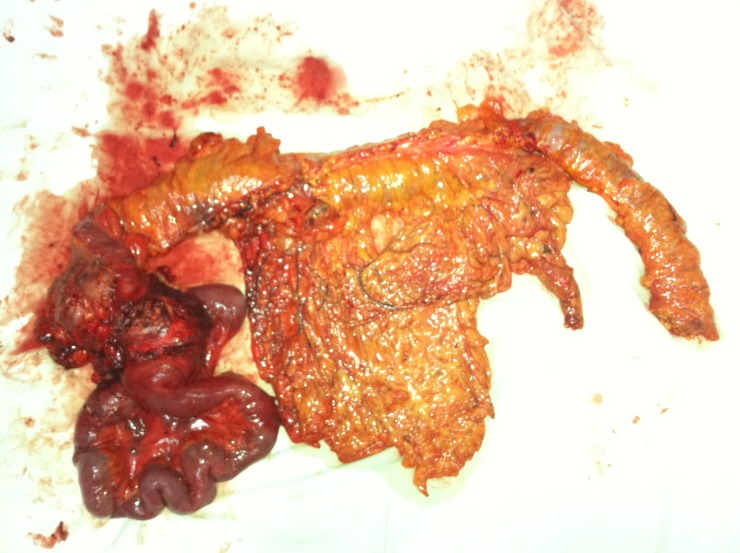
Subtotal colectomy specimen in a patient with invasive right colon tumor developed on familial adenomatous polyposis

A relatively recent option of using mechanic suture devices with a goal in digestive continuity reestablishment after removal of the intestine segment that carries the tumor is offering an increased degree of safety as well as a faster postoperative recovery [**12-15**]. These devices are represented by staplers of different types (EEA circular type, TA type, GIA type, endo-GIA type) and dimensions, allowing achievement of side-to-end, end-to-side or side-to-side anastomoses.

**Figure 5:**
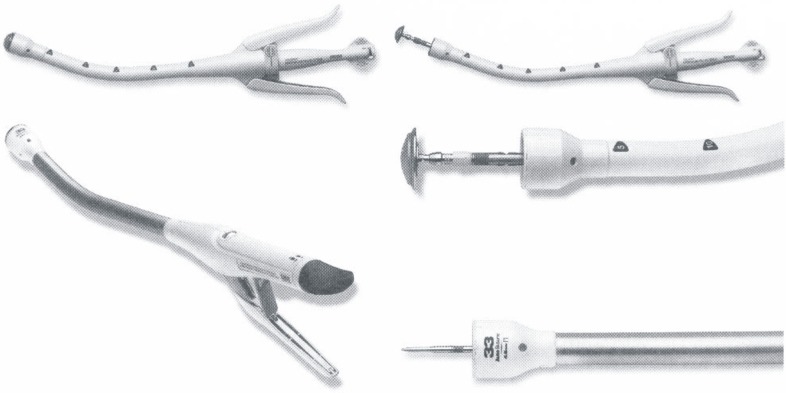
Intraluminal stapler (ILS), as known as end-to-end circular stapler (CEEA); also possible used for end-to-side, side-to-end or even side-to-side anastomosis

**Figure 6:**
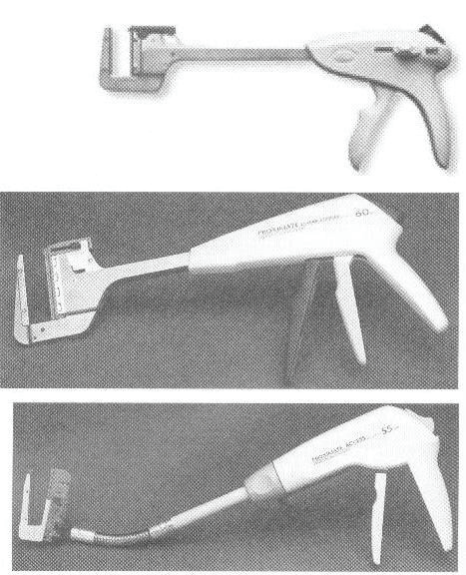
TA linear stapler (toracoabdominal or transversal anastomosis); used for closure of loop ends

**Figure 7:**
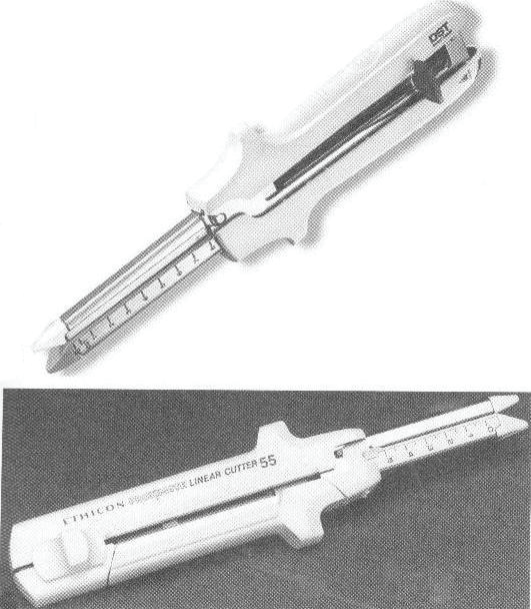
Gastrointestinal anastomosis stapler (GIA), used for side-to-side anastomosis; it includes a cutting knife

The laparoscopic technique, possible to be done in those cases of early stage colonic tumors, often combined with use of mechanic stapler devices, allowed such faster recovery of the patient, considering the much less operative trauma.

Fast-track surgery concept, introduced in colonic surgery by Danish surgeon Henrik Kehlet, starts gathering more and more adepts, its goal being that of better postoperative recovery, with evident benefits both for the patient and for the economic standards of the health system [**16**]. The fast-track surgery protocol presumes, after obtaining consent of the patient, the following attitude: normal diet in the day before operation, lack of mechanic preparation of the bowel, use of volatile anesthetic drugs with rapid induction, intraoperative restricted liquid intake, postoperative oxygen therapy, non-opioid postoperative analgesia, suppression of nasogastric tube at the end of the anesthesia and of the urinary probe in the first 24 postoperative hours, early postoperative feeding (liquids in first 6 hours, semi fluids after first day, normal from the second day), active mobilization of the patient since operative day.

Oncologic treatment supposes postoperative submission of most patients in chemotherapy programs, the mainly used regimen consisting in association of 5-FU with folinic acid.

Long-term results depend on tumor stage in diagnostic moment, the overall survival rate after 5 years being of around 45%. The follow up of the operated patients with colonic cancer presumes a clinical examination (every 3 months in the first 2 years and then every 6 months in the next 3 years), colonoscopy, and tumor markers determination and computer tomography (every 6 months in the first 2 years and then every year in the next 3 years).

## Material, Method, Results, Discussions

We realized an retrospective descriptive study lasting on the later 5 years (2007-2011), considering all cases of colonic cancer treated by surgical intervention in “Colentina” Clinical Surgery (307 patients), based on the analysis of medical papers and operatory registries.

Sex ratio was almost equal, with little predominance of the males (162 cases); as regards the provenience environment, such clear majority of the patients from urban sites could be seen, this being explained by the alimentary habits centered on predominant intake of animal protein diet.

Tumor topography: sigmoid colon - 112 cases, descending colon - 43 cases, transverse colon - 78 cases, cecum and ascending colon - 74 cases. The more frequent localization on left colon, mainly on sigmoid colon could be seen.

In 49 cases, the presentation was made in an emergency status, 39 cases being with acute intestinal occlusion and 10 cases with peritonitis by tumor perforation or by cecum diastatic perforation.

The clinical picture that brought the patient for medical help, except the cases presented in emergency, was carrying the following symptoms and signs (possible association of these):

-alterations of the intestinal transit, with increased constipation or constipation-diarrhea alter-nation: 187 cases;

-abdominal distension: 257 cases;

-abdominal pain: 113 cases;

-anemia of unknown reason: 97 cases;

-weight loss: 215 cases;

-digestive bleeding: 39 cases.

The diagnosis was mainly based, in the great majority of cases (287 cases), on colonoscopy with biopsy and histopathology examination.

The cases of our study group belonged to one of the following evolutionary stages:

- stage I: 34 cases;

- stage II: 75 cases;

- stage III: 135 cases;

- stage IV: 63 cases.

In the great majority of cases, the histopathological result indicated the existence of an adeno-carcinoma with different grades of differentiation.

Information needed for correct tumor staging was offered by investigations like abdominal echography, pulmonary radiography and abdominal computer tomography. The cases presented as emergencies benefited from diagnostic from plain abdominal radiography.

We started lately with such timidity to apply the principles of fast-track surgery, even trying to realize a mechanic preparation of the bowel anytime possible. Regarding the cases operated as emergencies, we practiced intraoperative cleaning of the bowel by washout technique.

The operations proceeded were represented by:

- right hemicolectomy: 86 cases;

- transverse segmental colectomy: 52 cases;

- left hemicolectomy: 48 cases;

- segmental sigmoidectomy: 112 cases;

- subtotal colectomy: 4 cases;

- total colectomy: 5 cases.

Considering the emergency operated cases, they benefited from the following interventions:

- those cases of occlusion by left colonic tumor and those with peritonitis by tumor perforation were solved in seriated interventions, the first time realizing the removal of the tumor with temporary colostomy, the second time for bowel continuity reestablishment being done 3 months later;

- those cases with peritonitis by diastatic cecum perforation were subjected for total colectomy with ileostomy as first operative time, followed 3 months later by reintervention for bowel continuity reestablishment (ileorectostomy).

We should mention here some cases with particular presentation and solution:

- 2 cases of appendical carcinoid that 1 month after the initial intervention (appendectomy), when the histopathological report was ready, needed reintervention for right hemicolectomy with ileotransversostomy;

- 13 cases with preexistent hepatic cirrhosis where the surgical intervention had to face with great hemorrhagic risks and the postoperative period was affected by decompensation of the hepatic function that had to be sustained;

- 5 cases of colonic tumor developed on familial adenomatous polyposis, submitted for subtotal colectomy (3 cases) or total colectomy (2 cases) with ileal pouch;

- 11 cases of splenic flexure colonic tumor with invasion in pancreatic tail and splenic hilum needed association of distal splenopancreatectomy;

- 3 cases of hepatic flexure colonic tumor with duodenal invasion needed reconstructive techniques for duodenum after tumor removal;

- 24 cases with 1-3 hepatic metastases associated wedge resections of the metastases.

In the last period, we started to use the stapler devices in colon-rectal cancer more and more. Thus, from the total of 307 patients operated for colon cancer, we reestablished the bowel continuity by side to end, end to side or side to side anastomoses using the staplers of EEA, TA, GIA or Endo-GIA types. The advantages were represented by shortened duration of the surgical intervention, with accomplishment of surgical sutures that have not produced any postoperative complication until now.

Surgical intervention was done in 5 cases by laparoscopic technique: right hemicolectomy - 2 cases, segmental sigmoidectomy - 2 cases, transversal colectomy - 1 case.

Postoperative complications occurred in 27 cases, being represented by:

- bleeding (3 cases),

- peritoneal abscesses: 5 cases,

- anastomotic fistula: 7 cases,

- wound infections: 12 cases;

11 cases need a surgical re-intervention.

Preoperative mortality was of 13 cases, due to one of the following complications:

- bronchopneumonia: 5 cases;

- IMA: 2 cases;

- hepatic failure: 3 cases;

- sepsis: 3 cases.

All the patients were subsequently submitted for oncologic treatment program with chemo¬therapy having the leading role.

Follow up of operated patients was done by clinical controls every 3 months in the first 2 years and then every 6 months in the next 3 years, colonoscopy, tumor markers and imagistic investigations done every 6 months in the first 2 years and then every year in the next 3 years.

## Conclusions

1. Incidence of colonic cancer in our country has an increasing tendency, probably due to the alignment of our culinary habits to those of the western civilization.

2. A majority proportion of cases discovered in advanced stages is still present, due to the persistence of poor sanitary education.

3. Colonic cancer prognostic mainly depends on the stage of disease, but also on the performance of a correct therapeutic protocol. It seems that for equivalent stages of disease, the tumors on right colon have worse prognostic than the left ones.

4. Surgical treatment represents the basic therapeutic link. In every case with invasion of the neighbor organs, it is important to realize a one-piece resection of the tumor mass.

5. The use of mechanic stapler devices allowed the shortening of the duration of the surgical interventions, with positive effect on postoperative recovery.

6. We are still reserved as regards the complete implementation of fast track surgery principles, but the even partial application of these encourages us to be more courageous in the future, considering the good results of centers with great experience in this domain.

7. Laparoscopic colonic surgery has to be proceed more and more in the future, the old reluctance based on studies showing risks for tumor dissemination being denied by the latest data published in scientific reviews. 

8. It is compulsory to submit all patients operated for colonic cancer to close periodic follow up with goal of early detection of local recurrences or distance metastases.
